# Reaction of Hydrogermanes ArGeH_3_ with Organolithium Reagents RLi: Unexpected Transfer of Organic Groups Instead of Lithiation

**DOI:** 10.1021/acs.inorgchem.6c01121

**Published:** 2026-04-30

**Authors:** Philipp Schmid, Paula Leuprecht, Anna-Maria Schaffler-Glössl, Vladimir Ya. Lee, Frank Uhlig

**Affiliations:** † Institute of Inorganic Chemistry, 27253 Graz University of Technology, Stremayrgasse 9/IV, 8010 Graz, Austria; ‡ Department of Chemistry, Institute of Pure and Applied Sciences, University of Tsukuba, Tsukuba, Ibaraki 305-8571, Japan

## Abstract

A series of novel triorganogermanes featuring an asymmetric germanium center were prepared by the straightforward alkylation of arylhydrogermanes with organolithium reagents (RLi). This synthetic approach represents a step-economical alternative to the traditionally employed procedure based on the quenching of germyllithium derivatives (prepared from the corresponding hydrogermanes) with organohalides. The appropriate choice of reaction solvent, temperature, and organolithium compound enables exclusive (or at least preferential) alkylation/arylation of organogermanium hydrides at the Ge–H bond (with RLi reacting as a nucleophile) at the expense of the traditionally anticipated lithiation of these compounds (with RLi reacting as a base).

## Graphical abstract

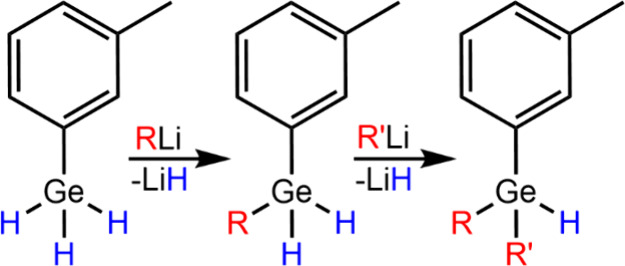

Alkylation of the C–H bond in organic compounds where the hydrogen atom is sufficiently acidic (terminal alkynes, allylic or benzylic derivatives, α-heteroatom-containing compounds, etc.) with organolithium reagents (RLi) is one of the major instruments in the toolbox of synthetic chemists commonly used to create new C–C bonds, one of the ultimate goals of organic chemistry.^1,2^ Typically, this is a two-step synthetic protocol involving initial lithiation, generating a new highly reactive organolithium intermediate (C–H + RLi → **C–Li** + RH), followed by the quenching of the latter with an appropriate electrophile (commonly, an alkyl halide R′X) to complete the alkylation sequence (C–Li + R′X → **C–R′** + LiX). Direct alkylation of organic compounds at the C–H bond with RLi (C–H + RLi → **C–R** + LiH) is generally not feasible due to the significant polarization of the C^δ−^–H^δ+^ bond toward the carbon atom caused by the C versus H electronegativity difference, unless the reactions are carried out under rather harsh conditions (e.g., decalin, 165 °C).^3^ The opposite is true for organosilanes: due to the inverted polarity of the Si^δ+^–H^δ−^ bond, alkyl- or arylsilanes are the expected products formed upon reaction of hydrosilanes with RLi.^4^

Lithiation of the Ge–H bonds of hydrogermanes with organolithium reagents (Ge–H + RLi → **Ge–Li** + RH) followed by quenching of the germyllithium intermediates with organohalides R′X (Ge–Li + R′X → **Ge–R′** + LiX) is one of the most popular methods for high-yielding preparation of organogermanium derivatives.^5,6^ Given the Ge/H electronegativity difference (small and inverted compared to that of the C/H pair),^7^ which is very similar to the case of the Si/H pair, and accordingly the higher propensity of the Ge–H bonds (compared to C–H bonds) to be polarized not only toward the Ge but also toward the H end, one can intuitively anticipate the possibility of the *direct* alkylation/arylation of hydrogermanes with organolithium reagents: Ge–H + RLi → **Ge–R** + LiH. However, despite the evident synthetic appeal of this straightforward one-step procedure, there are only a few publications reporting this behavior, mostly as chemical curiosities rather than synthetically valuable methods.^8–10^ Moreover, in none of these reports was alkylation the exclusive pathway, as preferential formation of the lithiated products was always observed.

Several years ago, we initiated a systematic study of trihydrogermanes RGeH_3_ [R = Ar (aryl), trialkylsilyl], their synthesis, structure, reactivity, and potential applications in materials science.^11–13^ In the course of the subsequent investigations, we found that the pathway of the reaction of aryltrihydrogermanes ArGeH_3_ with organolithium reagents RLi (R = alkyl, aryl) can be effectively and selectively directed toward a rather attractive pathway, namely, direct alkylation/arylation of the Ge–H bonds to form substituted derivatives ArGeH_*n*_R_3–*n*_ (*n* = 1, 2) instead of the traditionally anticipated lithiation of a Ge–H bond to form the germyllithium derivative (ArGeH_2_Li). By this step-economical approach, (*p*-tolyl)­GeH_2_R (R = ^*n*^Bu, ^*t*^Bu) were prepared as a proof of concept, but more thorough research beyond this preliminary result was out of the scope of our previous publication.^12^ Below we report a systematic investigation of the ArGeH_3_/RLi reaction system, along with a discussion of the key factors (solvents, temperature, nature of Ar and R substituents) that influence the overall reaction outcome.

The reaction of (*m*-tolyl)­trihydrogermane ((*m*-tolyl)­GeH_3_)^14^ with organolithium reagents (RLi, R = ^*i*^Bu, Ph) in THF at −25 °C preferentially proceeds as a lithiation process, in full accord with general expectations, primarily generating the corresponding germyllithium derivative (*m*-tolyl)­GeH_2_Li (**1**), formation of which was confirmed by quenching with ^*t*^BuMe_2_SiCl (Scheme 1A). While small amounts of (*m*-tolyl)­GeH_2_R and (*m*-tolyl)­GeHR_2_ (R= ^*i*^Bu, Ph) were also formed in THF, this may be caused by the use of commercially obtained organolithium reagents, which introduce additional solvents into the mixture (^*i*^BuLi 1.7 M in ^*n*^heptane, PhLi 1.9 M in ^*n*^Bu_2_O). In sharp contrast, the same reaction carried out in diethyl ether at −25 °C resulted in the exclusive formation of the alkylated products (*m*-tolyl)­GeH_2_R (R = ^*n*^Bu (**2a**); R = ^*i*^Bu (**2b**); R= Ph (**2c**)), which were isolated by fractional recondensation in moderate yields of 55% (for **2a**), 45% (for **2b**), and 45% (for **2c**) (Scheme 1B). The use of the more polar, stronger coordinating THF favors the lithiation pathway, in which RLi primarily acts as a base attacking the Ge–**H** proton, whereas the much less polar diethyl ether promotes the alkylation pathway, in which RLi behaves as a nucleophile attacking the **Ge**–H germanium center.

**Scheme 1:**
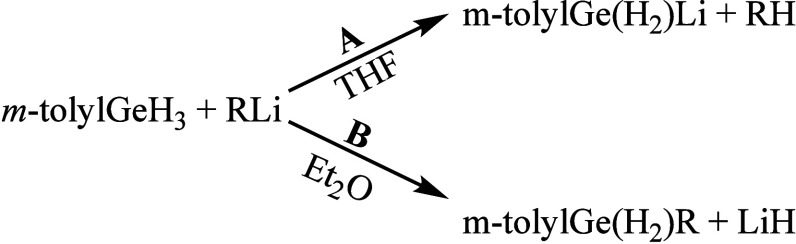
Reactions of (*m*-Tolyl)­GeH_3_ with RLi: (A) Lithiation in THF; (B) Alkylation in Et_2_O

Alkylation/arylation of hydrogermanes is not without precedents. Gilman and Gerow reported formation of minor amounts of Ph_3_Ge-GePh_3_ upon reflux of the reaction mixture (Ph_3_GeH + Ph_3_GeLi) in ethylene glycol dimethyl ether or diethyl ether.^8^ However, in ethereal solvents (moreover under reflux conditions), it is very likely that the formation of Ph_3_Ge–GePh_3_ involves an electron-transfer event rather than the nucleophilic substitution process. Cross and Glockling discussed some reactions of benzylgermanes forming “alkylation” products, but identification of the latter compounds is not convincing, given the lack of spectroscopic data.^9^ The most relevant comparison to our results can be done with the work of Castel and co-workers,^10^ who reported competing lithiation and alkylation of PhGeH_3_ in THF with ^*t*^BuLi to form PhGeH_2_Li (69%), PhGeH_2_(^*t*^Bu) (9%), and PhGeH­(^*t*^Bu)_2_ (8%) but did not attempt to isolate the pure compounds from the product mixture.

Apart from the predominating solvent effect, the steric demand of both the Ar-substituent at Ge in the ArGeH_3_ substrate and the R-group in the RLi organolithium reagent plays a decisive role in directing the reaction pathway toward either lithiation or alkylation/arylation. If *m*-tolylGeH_3_ or *p*-tolylGeH_3_ are employed, the formation of the nucleophilic substitution products ArGeH_2_R is favored over the lithiated products ArGeH_2_Li. In contrast, use of the notably sterically bulkier *o*-tolyl, 2,6-dimethylphenyl, and mesityl groups in ArGeH_3_ results in significantly higher—and under some conditions even exclusive—conversion into the germyllithium derivatives, as shown in our earlier work.^15^ An identical observation was reported by Castel and co-workers, who found both lithiation and alkylation pathways in the reaction of ^*t*^BuLi with PhGeH_3_ resulting in a mixture of PhGeH_2_Li and PhGeH_2_(^*t*^Bu), whereas in the reaction of ^*t*^BuLi with the bulkier MesGeH_3_ (Mes = 2,4,6-trimethylphenyl) only lithiation was observed, forming MesGeH_2_Li.^10^

An increase in the steric shielding around germanium in ArGeH_3_ (on going from *p*-tolyl to *m*-tolyl to *o*-tolyl to 2,6-dimethylphenyl and to mesityl) blocks the back-side attack of the R^–^ carbanion on the Ge center (alkylation pathway) and at the same time promotes the reaction of R^–^ with the Ge–**H** terminal proton (lithiation pathway). The influence of the size of the R substituent in RLi also goes in the same direction, with sterically bulky R groups such as ^*t*^Bu increasing the rate of lithiation over smaller R groups such as Me.^15^ However, one should not rule out the influence of the basicity of RLi reagents, which can often act in cooperation with the steric effect (very bulky and strongly basic ^*t*^BuLi) but sometimes counteract the influence of the steric bulk (relatively large but weakly basic PhLi): Me < ^*n*^BuLi < ^*i*^BuLi < *sec*-BuLi ∼ PhLi < ^*t*^BuLi (bulkiness order) versus PhLi < MeLi < ^*n*^BuLi ∼ ^*i*^BuLi < *sec*-BuLi < ^*t*^BuLi (basicity order).

The influence of the temperature factor on the reaction outcome (that is, alkylation versus lithiation) was studied for the reaction of (*m*-tolyl)­GeH_3_ with ^*i*^BuLi and PhLi in the following temperature range: room temperature (RT) → 0 °C → −15 °C → −25 °C (see Table 1). In the reaction of *m*-tolylGeH_3_ with ^*i*^BuLi in Et_2_O, lower temperatures (−15 °C, −25 °C) clearly favor alkylation at Ge, resulting in the formation of **2b** as the only identified product. At 0 °C, **2b** still presents as a major product (80%) along with unreacted starting material (5%), although the lithiation product **1** (identified as its ^*t*^BuMe_2_SiCl quenching product) is also formed in a notable amount (14%). Further increase of the reaction temperature to ambient conditions results in nonselective formation of a mixture of **2b** (41%), **1** (34%), the double alkylation product (*m*-tolyl)­GeH­(^*i*^Bu)_2_ (14%), and unreacted (*m*-tolyl)­GeH_3_ (11%). In the reaction of (*m*-tolyl)­GeH_3_ with PhLi in Et_2_O, lower temperatures also promote alkylation at germanium (92% at −25 °C), whereas at higher temperatures double alkylation to (*m*-tolyl)­GeHPh_2_ becomes a notable reaction pathway (4% → 20% at −25 °C → RT). To prove the importance of the solvent effect, reactions at −25 °C were also performed in THF. This resulted in the formation of germyllithium species as primary products, whereas no lithiation whatsoever was observed in Et_2_O at this temperature.

**Table 1:** Product Distributions in the Reactions of *m*-TolylGeH_3_ with RLi (R = ^*i*^Bu, Ph) in Et_2_O or THF at the Specified Temperatures

	reaction with ^*i*^BuLi (1.7 M in *n*-heptane)					reaction with PhLi (1.9 M in ^*n*^Bu_2_O)				
	in Et_2_O				in THF	in Et_2_O				in THF
compound	RT	0 °C	–15 °C	–25 °C	–25 °C	RT	0 °C	–15 °C	–25 °C	–25 °C
*m*-tolylGeH_3_	11	5	0	1	2	9	11	7	3	5
*m*-tolyl**GeH**_**2**_ **(R)**	**41**	**80**	**100**	**99**	**19**	**52**	**70**	**79**	**92**	**32**
*m*-tolylGeH_2_(Li)	34	14	0	0	74	14	12	0	0	51
*m*-tolylGeHR_2_	14	1	0	0	3	20	6	14	4	7
*m*-tolylGeH(R)Li	0	0	0	0	2	5	1	0	0	5

With two remaining H atoms, the nucleophilic substitution products (*m*-tolyl)­GeH_2_R are obvious targets for subsequent reaction with another organolithium reagent R′Li to form the double substitution products (*m*-tolyl)­GeHRR′. Given the increase in steric crowding around the Ge center in (*m*-tolyl)­GeH_2_R (compared to that of the starting (*m*-tolyl)­GeH_3_), the appropriate choice of R′Li is important. The reaction of the dihydrides **2a** and **2b** with MeLi in diethyl ether at 0 °C resulted in the clean formation of the monohydrides (*m*-tolyl)­GeHR­(Me) (R = ^*n*^Bu (**3a**); R = ^*i*^Bu (**3b**)), isolated by fractional recondensation in satisfactory yields of 57% (for **3a**) and 59% (for **3b**). The second alkylation was also possible with butyllithium reagents: **2b** reacted with ^*n*^BuLi to form the double alkylation product (*m*-tolyl)­GeH­(^*i*^Bu)­(^*n*^Bu) **3c** (38%) with two different butyl substituents, and the same compound was prepared by the reaction of **2a** with ^*i*^BuLi (30% yield). Work on the substitution of the last remaining hydrogen in triorganogermanes **3a**–**c** to achieve persubstituted tetraorganogermanes (*m*-tolyl)­Ge­(R)­(R′)­(R″) is currently underway in our laboratories.

Triorganogermanes **3a**–**c** feature a Ge atom with four different substituents. Racemic/chiral organogermanes featuring an asymmetric Ge center are typically prepared in three common ways: (1) by a stepwise procedure via a repetitive lithiation–alkylation reaction sequence Ge–H + RLi → Ge–Li + R′X → Ge–R′ (traditional method); (2) through hydrogermylation of alkenes, i.e., RR′GeH_2_ + H_2_C=CH–R″ → RR′Ge­(H)–CH_2_–CH_2_–R″ (a novel method);^16^ and (3) via carbene insertion of dihydrogermanes, i.e., RR′GeH_2_ + N_2_CAr_2_ → RR′Ge­(H)–CHAr_2_ (a novel method).^17^ Compared to the most used method (1), our approach, based on the direct alkylation of hydrogermanes with organolithium reagents and thus the absence of the intermediate step of germyllithium derivatives, represents a simpler and more effective alternative for the synthesis of asymmetric organogermanes. They could potentially be optically resolved to pure enantiomeres via reported methods,^18,19^ which might find industrial applications.

In conclusion, we developed a simple protocol for the direct alkylation/arylation of hydrogermanes with commercially available organolithium reagents that can be applied as a synthetically feasible preparative procedure on the way to poly- and persubstituted organogermanes (Scheme 2).

**Scheme 2:**
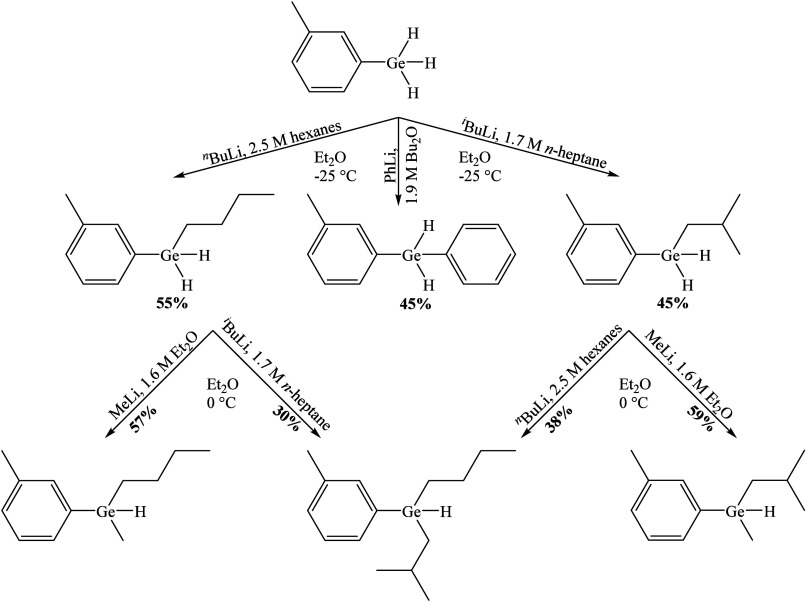
Substituted Organogermanes Prepared in This Work

## Supplementary Material



## Notes

Experimental section and NMR spectral charts (PDF)
