# Supplementary material for: Reaction of Hydrogermanes ArGeH3 with Organolithium Reagents RLi: Unexpected Transfer of Organic Groups Instead of Lithiation
Source: Inorg Chem. 2026 Apr 30;65(18):9746–9. doi: 10.1021/acs.inorgchem.6c01121 (PMC13169375; doi:10.1021/acs.inorgchem.6c01121)
Supplement: Supplementary file 1 [file ic6c01121_si_001.pdf]

SUPPORTING INFORMATION

Reaction of Hydrogermanes $ArGeH_3$ with Organolithium Reagents RLi: Unexpected Transfer of Organic Groups Instead of Lithiation

Philipp Schmid,[1] Paula Leuprecht,[1] Anna-Maria Schaffler-Glössl,[1] Vladimir Ya. Lee*[2] and Frank Uhlig*[1]

[1]P. Schmid, P. Leuprecht, A.-M. Schaffler-Glössl, F. Uhlig
Institute of Inorganic Chemistry, Graz University of Technology, Stremayrgasse 9/IV, 8010 Graz, Austria

[2]V. Ya. Lee
Department of Chemistry, Institute of Pure and Applied Sciences, University of Tsukuba, Tsukuba 305-8571, Ibaraki, Japan

*To whom the correspondence should be addressed:
Vladimir Ya. Lee, leevya@chem.tsukuba.ac.jp
Frank Uhlig, frank.uhlig@tugraz.at

Contents of the Supporting Information:

1. Experimental Section: (a) general procedures; (b) experimental procedures and spectroscopic data for compounds **2a-c** and **3a-c**; (c) ^{1}H- and ^{13}C{^{1}H} NMR spectral charts for compounds **2a-c** and **3a-c** (Figures S1–S12) S2–S12

2. References: S13

1. Experimental Section.

General procedures. All experimental manipulations were performed using high-vacuum line or Schlenk techniques. All ethereal and hydrocarbon solvents were predried using "Innovative Technology Inc." solvent system. THF was additionally distilled over $LiAlH_4$ immediately prior to use. NMR spectra were recorded on a Jeol ECZ 400 with Royalprobe^{TM} HFX (^{1}H NMR at 400.1 MHz, ^{13}C NMR at 100.6 MHz) NMR spectrometer. High-resolution mass spectra were measured on a Jeol JMC-T2000GC (AccuTOFTM GC-Alpha) mass spectrometer equipped with a 8890 GC system (column: DB-5MS, 30 m × 0.25 mm × 0.25 μm) and a 7693A autosampler from Agilent, applying electron ionization EI^+ mode (70 eV, 200μA, source at 250 °C). Product mixtures from temperature studies were analyzed on an Agilent Technologies 7890A gas chromatograph (column: HP5, 30 m × 0.25 mm × 0.25 μm), manual injection at 250 °C, Agilent Technologies 5975 C mass detector in full scan mode (50–500 m/z). Elemental analysis was performed on an Elementar Vario EL III machine. Starting *m*-tolylGeH_3 was prepared was prepared according to the published procedure.[1] All other starting materials were obtained commercially and used without further purifications.

Caution! *Organolithium reagents are highly pyrophoric. All manipulations of these compounds were performed on a small scale under argon atmosphere using proper needle and syringe techniques.*

(a) Experimental procedure and spectroscopic data for ***(m-tolyl)GeH$_2$(^{n}Bu) 2a***.

A Schlenk tube (50 mL) equipped with a glass stir bar was charged with *m*-tolylGeH_3 (1.07 g, 6.40 mmol) in Et_2O (4 mL). After cooling to –25 °C (EtOH/liquid N_2), ^{n}BuLi (2.5 M solution in hexanes, 6.5 mmol) was added dropwise, and the mixture was stirred overnight while slowly allowing it to warm to room temperature. After quenching with H_2O (5 mL), phases were separated, and the aqueous phase extracted twice with Et_2O (3 × 5 mL). The combined organic phases were dried (Na_2SO_4), filtered, volatiles were removed (~ 0.1 mbar, room temperature), and the product **2a** was isolated through recondensation (1.2 × 10^{-3} mbar, 40 °C) as clear colorless liquid (0.79 g, 55%). ^{1}H NMR (C_6D_6) δ 0.82 (t, ^3J = 7.35 Hz, 3 H, CH_3), 1.03–1.09 (m, 2 H, CH_2), 1.23–1.32 (m, 2 H, CH_2), 1.38–1.47 (m, 2 H, CH_2), 2.13 (s, 3 H, Ar-CH_3), 4.55 (t, ^3J = 2.86 Hz, 2 H, GeH_2), 7.00 (d, ^3J = 7.42 Hz, 1 H, H_{Ar}), 7.12 (m, 1 H, H_{Ar}), 7.31 (d, ^3J = 9.06 Hz, 2 H, H_{Ar}). ^{13}C NMR (C_6D_6, 101 MHz) δ 11.8, 13.9, 21.5, 26.0, 29.3, 128.5, 129.8,

132.3, 135.2, 136.0, 137.7. HRMS (EI): *m/z* calcd. for $C_{11}H_{18}Ge$ 224.06148 $[M]^+$, found 224.06124. Anal. Calcd. for $C_{11}H_{18}Ge$: C, 59.27; H, 8.14. Found: C, 59.10; H, 7.92.

(b) Experimental procedure and spectroscopic data for ***(m-tolyl)GeH$_2$(^{i}Bu) 2b****.*

A Schlenk tube (50 mL) equipped with a glass stir bar was charged with *m*-tolylGeH$_3$ (1.04 g, 6.26 mmol) in Et_2O (4 mL). After cooling to –25 °C (EtOH/liquid N_2), ^{i}BuLi (1.7 M solution in ^{n}heptane, 6.5 mmol) was added dropwise, and the mixture was stirred overnight while slowly allowing it to warm to room temperature. After quenching with H_2O (10 mL), phases were separated, and the aqueous phase extracted more with Et_2O (2 × 5 mL). The combined organic phases were dried (Na_2SO_4), filtered, volatiles were removed (~ 0.1 mbar, room temperature), and the product **2b** was isolated through recondensation (8.1 × 10^{-4} mbar, room temperature) as clear colorless liquid (0.62 g, 45%). ^{1}H NMR (C_6D_6) δ 0.92 (d, ^3J = 6.63 Hz, 6 H, 2 CH_3), 1.04–1.08 (m, 2 H, CH_2), 1.73–1.84 (m, 1 H, CH), 2.12 (s, 3 H, Ar-CH_3), 4.57 (t, ^3J = 3.31 Hz, 2 H, GeH_2), 7.00 (d, ^3J = 7.47 Hz, 1 H, H_{Ar}), 7.13 (t, ^3J = 7.40 Hz, 1 H, H_{Ar}), 7.32 (d, ^3J = 10.38 Hz, 2 H, H_{Ar}). ^{13}C NMR (C_6D_6, 101 MHz) δ 21.4, 22.6, 25.3, 27.0, 128.5, 129.8, 132.3, 135.5, 136.0, 137.7. HRMS (EI): *m/z* calcd. for $C_{11}H_{18}Ge$ 224.06148 $[M]^+$, found 224.06047. Anal. Calcd. for $C_{11}H_{18}Ge$: C, 59.27; H, 8.14. Found: C, 59.15; H, 7.81.

(c) Experimental procedure and spectroscopic data for ***(m-tolyl)GeH$_2$(Ph) 2c****.*

A Schlenk tube (50 mL) equipped with a glass stir bar was charged with *m*-tolylGeH$_3$ (1.75 g, 11.47 mmol) in Et_2O (12 mL). After cooling to –25 °C (EtOH/liquid N_2), PhLi (1.9 M solution in ^{n}Bu$_2$O, 11.4 mmol) was added dropwise, and the mixture was stirred overnight while slowly allowing to warm to room temperature. After quenching with H_2O (10 mL), phases were separated, and the aqueous phase extracted twice with Et_2O (2 × 5 mL). The combined organic phases were dried (Na_2SO_4), filtered, volatiles were removed (~ 0.1 mbar, room temperature), and the product **2c** was isolated through fractional recondensation (9.0 × 10^{-4} mbar, 90 °C) as clear colorless liquid (1.25 g, 45%). ^{1}H NMR (C_6D_6) δ 2.05 (s, 3 H, Ar-CH_3), 5.20 (s, 2 H, GeH_2), 6.99 (d, ^3J = 7.50 Hz, 1 H, H_{Ar}), 7.15–7.07 (m, 4 H_{Ar}), 7.15–7.07 (m, 4 H, H_{Ar}), 7.32 (d, ^3J = 7.11 Hz, 2 H, H_{Ar}), 7.45-7.50 (m, 2 H, H_{Ar}). ^{13}C NMR (C_6D_6) δ 21.3, 127.5, 128.6, 128.7, 129.1, 129.3, 130.2, 132.6, 134.0, 134.5, 135.5, 136.2, 138.0. HRMS (EI): *m/z* calcd. for

$C_{13}H_{13}Ge$ 244.03017 $[M]^+$, found 244.02939. Anal. Calcd. for $C_{13}H_{14}Ge$: C, 64.29; H, 5.81. Found: C, 64.38; H, 5.65.

(d) Experimental procedure and spectroscopic data for ***(m-tolyl)GeH(^{n}Bu)Me 3a***.

A Schlenk tube (50 mL) equipped with a glass stir bar was charged with **2a** (461 mg, 2.07 mmol) in Et_2O (2.5 mL). After cooling to 0 °C, MeLi (1.6 M solution in Et_2O, 2.08 mmol) was added. The cooling bath was removed and the reaction mixture was stirred overnight at room temperature. After quenching with H_2O (3 mL), phases were separated, and the aqueous phase extracted twice with Et_2O (2 × 5 mL). The combined organic phases were dried (Na_2SO_4), filtered, volatiles were removed (~ 0.1 mbar, room temperature), and the product **3a** was isolated through recondensation (9.1 × 10^{-4} mbar, 40 °C) as clear colorless liquid (0.28 g, 57%). ^{1}H NMR (C_6D_6) δ 0.40 (d, ^3J = 2.73 Hz, 3 H, CH_3), 0.84 (t, ^3J = 7.24 Hz, 3 H, CH_3) 0.92–1.07 (m, 2 H, α-CH_2), 1.25–1.34 (m, 2 H, CH_2), 1.38–1.46 (m, 2 H, CH_2), 2.16 (s, 3 H, Ar-CH_3), 4.59–4.64 (m, 1 H, GeH), 7.02 (d, ^3J = 7.48 Hz, 1 H, H_{Ar}), 7.16 (m, 1 H, H_{Ar}), 7.30 (d, ^3J = 7.25 Hz, 1 H, H_{Ar}), 7.34 (s, 1 H, H_{Ar}). ^{13}C NMR (C_6D_6, 101 MHz) δ –6.0, 14.0, 14.4, 21.5, 26.4, 28.4, 128.4, 129.7, 131.5, 135.1, 137.5, 138.9. HRMS (EI): *m/z* calcd. for $C_{12}H_{20}Ge$ 238.07733 $[M]^+$, found 238.07700. Anal. Calcd. for $C_{12}H_{20}Ge$: C, 60.81; H, 8.51. Found: C, 60.91; H, 8.64.

(e) Experimental procedure and spectroscopic data for ***(m-tolyl)GeH(^{i}Bu)Me 3b***.

A Schlenk tube (50 mL) equipped with a glass stir bar was charged with **2b** (470 mg, 2.11 mmol) in Et_2O (2.5 mL). After cooling to 0 °C, MeLi (1.6 M solution in Et_2O, 2.24 mmol) was added. The cooling bath was removed and the reaction mixture was stirred overnight at room temperature. After quenching with H_2O (2 mL), phases were separated, and the aqueous phase extracted twice with Et_2O (2 × 2.5 mL). The combined organic phases were dried (Na_2SO_4), filtered, volatiles were removed (~ 0.1 mbar, room temperature), and the product **3b** was isolated through recondensation (9.8 × 10^{-4} mbar, 40 °C) as clear colorless liquid (0.30 g, 59%). ^{1}H NMR (C_6D_6) δ 0.41 (d, ^3J = 2.78 Hz, 3 H, CH_3), 0.88–1.08 (m, 5 H, CH_2 and 2 CH_3), 1.75–1.85 (m, 1 H, CH), 2.15 (s, 3 H, Ar-CH_3), 4.63–4.69 (m, 1 H, GeH), 7.02 (d, ^3J = 7.46 Hz, 1 H, H_{Ar}), 7.13–7.19 (m, 1 H, H_{Ar}), 7.31 (d, ^3J = 7.24 Hz, 1 H, H_{Ar}), 7.35 (s, 1 H, H_{Ar}). ^{13}C NMR (C_6D_6, 101 MHz) δ –5.1, 21.5, 25.5, 25.7, 26.6, 128.4, 129.6, 131.5, 135.1, 137.6, 139.3. HRMS

(EI): *m/z* calcd. for $C_{12}H_{20}Ge$ 238.07733 $[M]^+$, found 238.07696. Anal. Calcd. for for $C_{12}H_{20}Ge$: C, 60.81; H, 8.51. Found: C, 61.04 H, 8.66.

(f) Experimental procedure and spectroscopic data for ***(m-tolyl)GeH(^{i}Bu)^{n}Bu 3c***.

A Schlenk tube (50 mL) equipped with a glass stir bar was charged with 2b (165 mg, 0.738 mmol) in Et2O (1.0 mL). After cooling to –25 °C (EtOH/liquid N2), nBuLi (2.5 M solution in hexanes, 0.75 mmol) was added dropwise. The reaction mixture was stirred over the weekend while slowly allowing it to warm to room temperature. After quenching with H2O (5 mL), more Et2O (2 mL) was added to facilitate phase separation. The aqueous phase was extracted twice with Et2O (2 x 3 mL), and the combined organic phases were dried (Na2SO4) and filtered. Solvents were removed under vacuum (0.1 mbar, room temperature) and then remaining starting material under vacuum on heating (9.13 × 10–4 mbar, 40 °C). The product 3c was isolated through recondensation (9.01 × 10–4 mbar, 100 °C) as clear colorless liquid (0.078 g, 38%).
Alternatively, **3c** was also produced by an identical procedure from **2a** (295 mg, 1.32 mmol) in Et_2O (1.0 mL) with ^{i}BuLi (1.7 M solution in ^{n}heptane, 1.36 mmol) to yield 110 mg (30%) of product.
^{1}H NMR (C_6D_6) δ 0.85 (t, ^3J = 7.30 Hz, 3 H, CH_3), 0.97 (t, ^3J = 6.27 Hz, 6 H, 2 CH_3), 1.01–1.12 (m, 4 H, 2 CH_2), 1.27–1.37 (m, 2 H, CH_2), 1.42–1.52 (m, 2 H, CH_2), 1.80–1.90 (m, 1 H, CH), 2.16 (s, 3 H, CH_3), 4.67 (quint, ^3J = 3.07 Hz, 1 H, GeH), 7.03 (d, ^3J = 7.57 Hz, 1 H, H_{Ar}), 7.18 (d, ^3J = 7.47 Hz, 1 H, H_{Ar}), 7.35 (d, ^3J = 7.24 Hz, 1 H, H_{Ar}), 7.40 (s, 1 H, H_{Ar}). ^{13}C NMR (C_6D_6, 101 MHz) δ 13.9, 13.9, 21.5, 24.3, 25.7, 25.8, 26.4, 26.7, 28.7, 129.6, 131.8, 135.6, 137.6, 138.8. HRMS (EI): *m/z* calcd. for $C_{15}H_{26}Ge$ 280.12434 $[M]^+$, found 280.12425 Anal. Calcd. for $C_{15}H_{26}Ge$: C, 64.57; H, 9.39. Found: C, 64.81; H, 9.35.

(g) Experimental procedure for temperature studies.

A Schlenk tube (70 mL) equipped with a glass stir bar was charged with *m*-tolylGeH_3 (0.5 mL, ~3.6 mmol). Et_2O or THF (1.6 mL) was added and the flask was put into an appropriate bath (room temperature water bath, ice bath or cryostat). The flask was kept in the bath for 30 minutes before the next step to allow for equilibration. One equivalent of ^{i}BuLi (1.7M in

^{n}heptane, 2.1 ml) or PhLi (1.9 M in $^{n}Bu_2O$, 1.9 ml) was added and the flask was shaken for proper mixing. The flask was kept in the bath overnight. The next day, the flask was removed from the temperature bath, and $^{t}BuMe_2SiCl$ (1 M solution in THF, 3.6 mL) was added to convert resulting germyllithium salts to the corresponding ($^{t}BuMe_2Si$)Ge-derivatives for their GC–MS analysis. Formed inorganic salts were removed by extraction with water, the organic phase was dried (Na_2SO_4), filtered, Et_2O and THF were removed under vacuum (~10 mbar, room temperature). The residue was analyzed with GC–MS machine. The reported percentages are the relative peak area of all detected germanium-containing species.

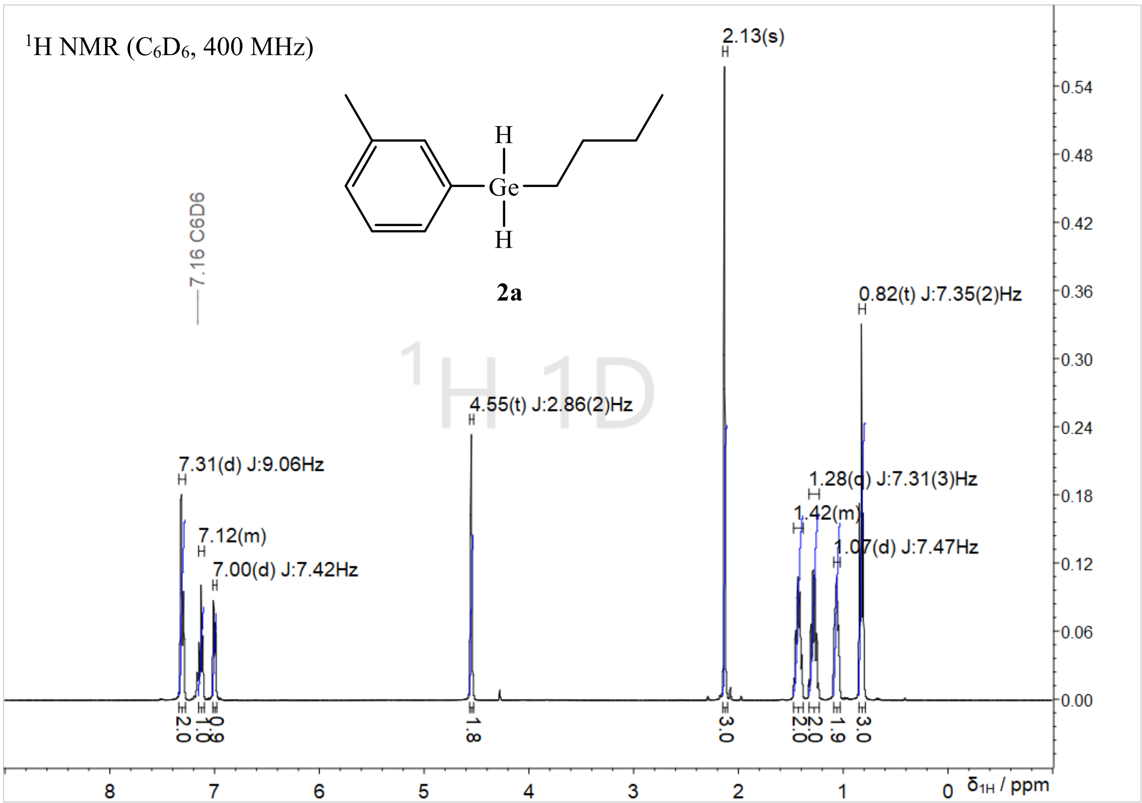

Figure S1. ^{1}H NMR spectrum of **2a** (C$_6$D$_6$).

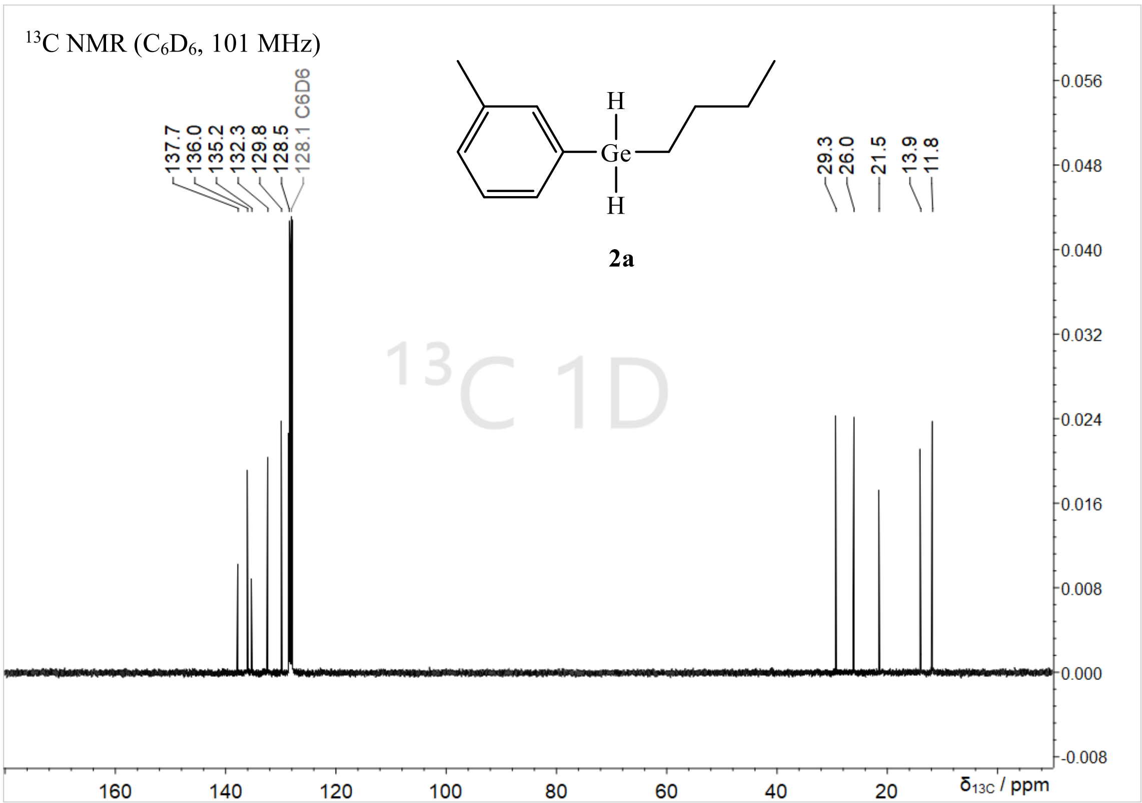

Figure S2. ^{13}C{^{1}H} NMR spectrum of **2a** (C$_6$D$_6$).

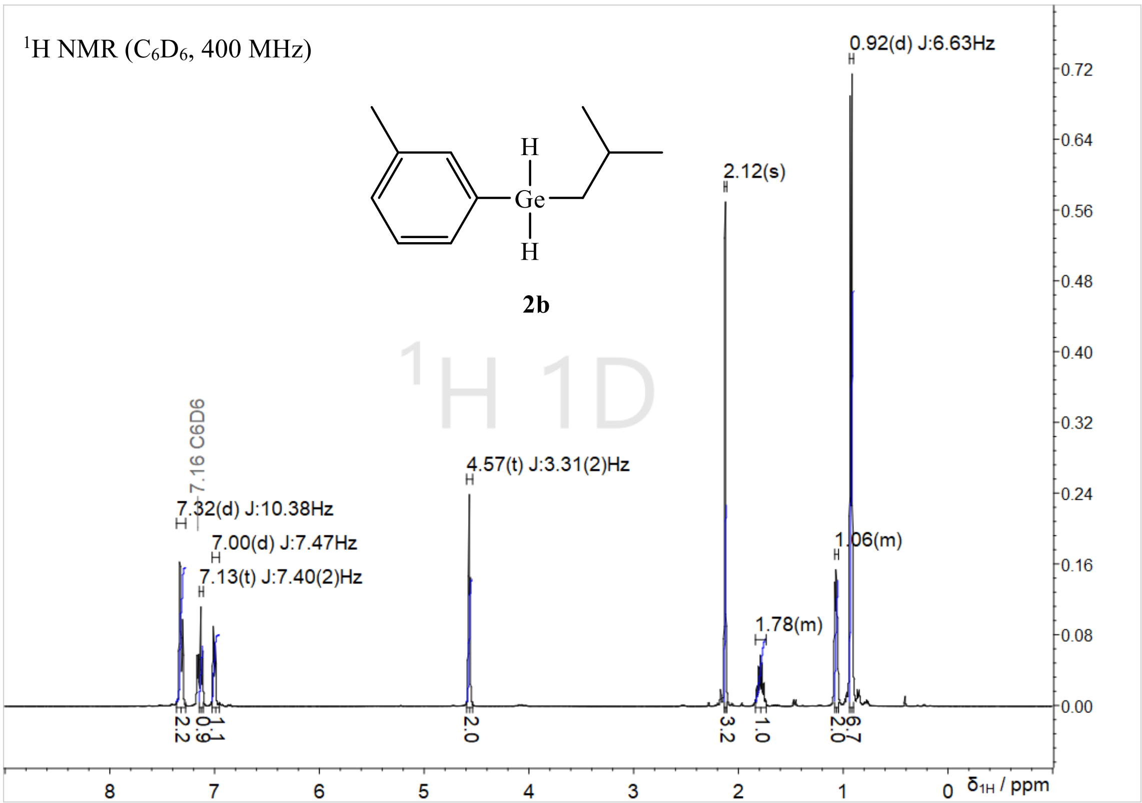

Figure S3. ^{1}H NMR spectrum of **2b** (C_6D_6).

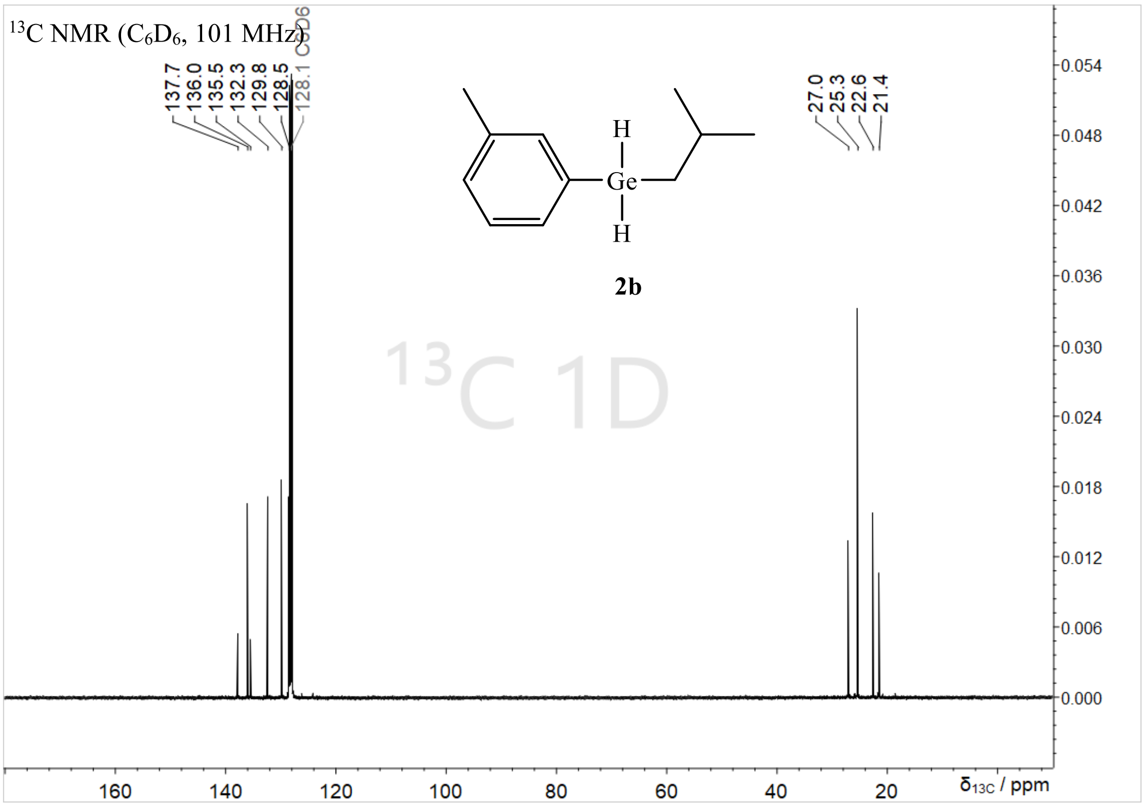

Figure S4. ^{13}C{^{1}H} NMR spectrum of **2b** (C_6D_6).

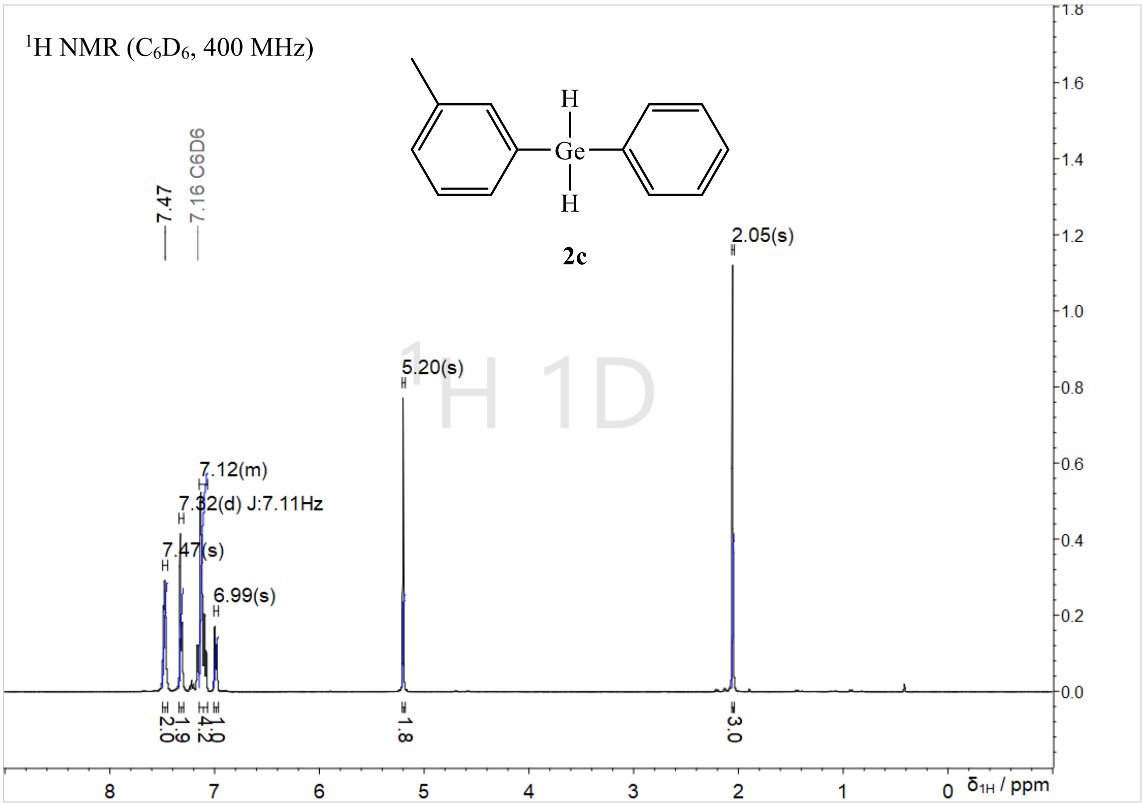

Figure S5. ¹H NMR spectrum of (m-tolyl)GeH_2(Ph) **2c** (C_6D_6).

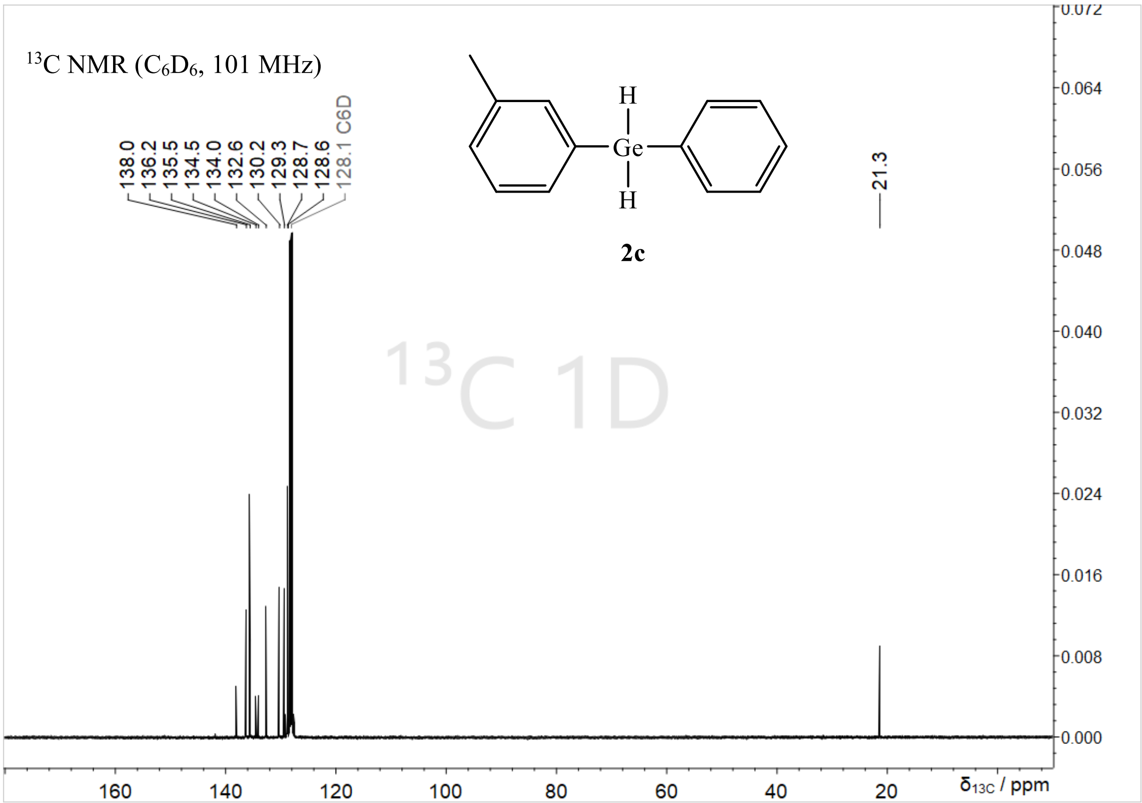

Figure S6. ¹³C{¹H} NMR spectrum of (m-tolyl)GeH_2(Ph) **2c** (C_6D_6).

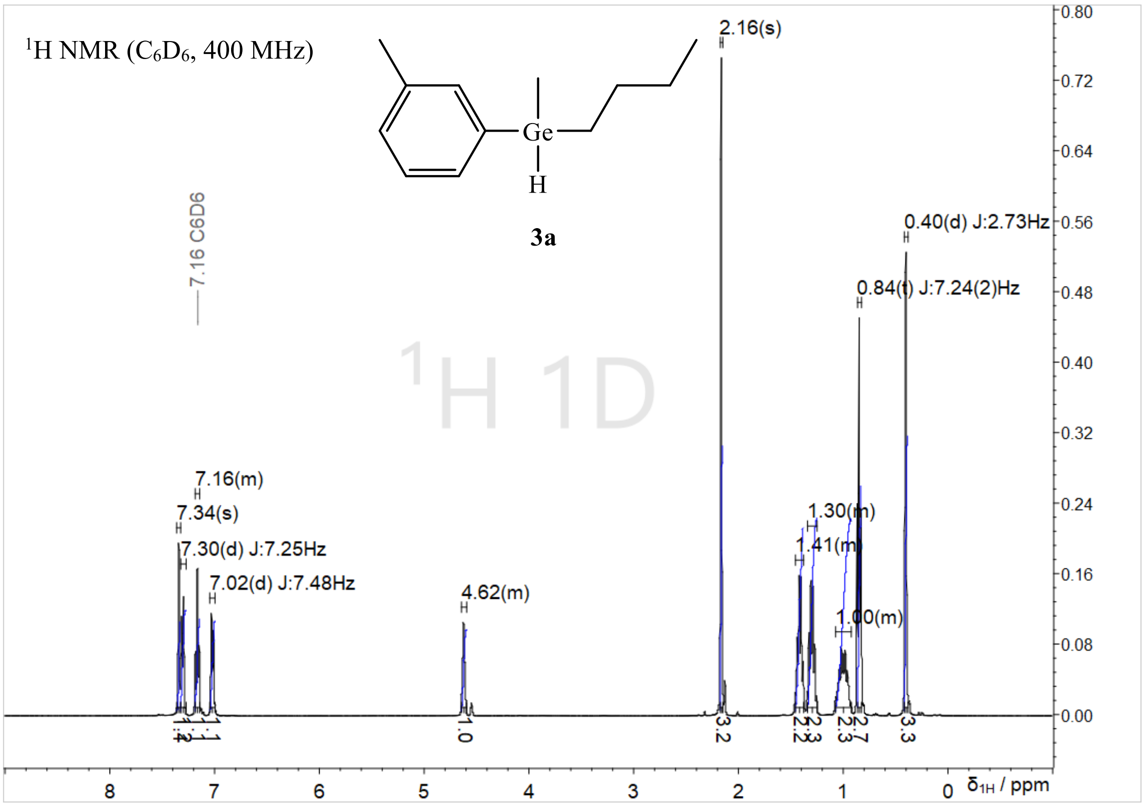

Figure S7. ^{1}H NMR spectrum of **3a** **(**C_6D_6).

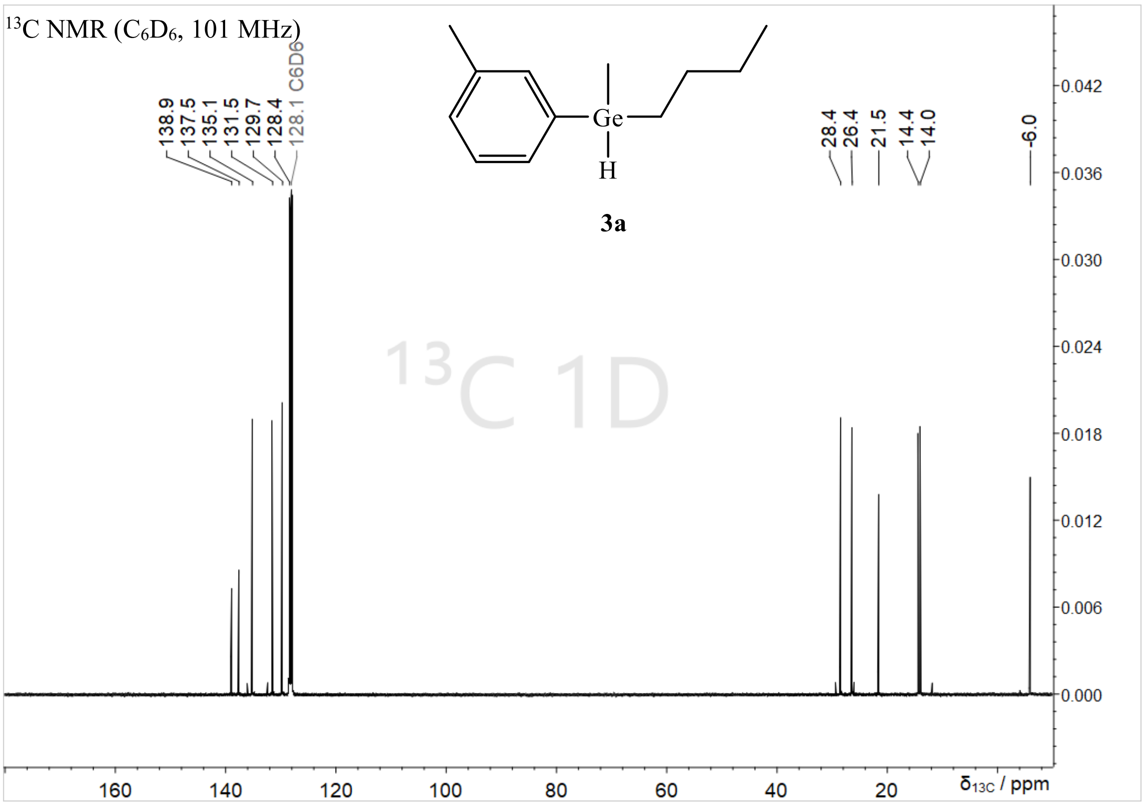

Figure S8. ^{13}C{^{1}H} NMR spectrum of **3a** (C_6D_6).

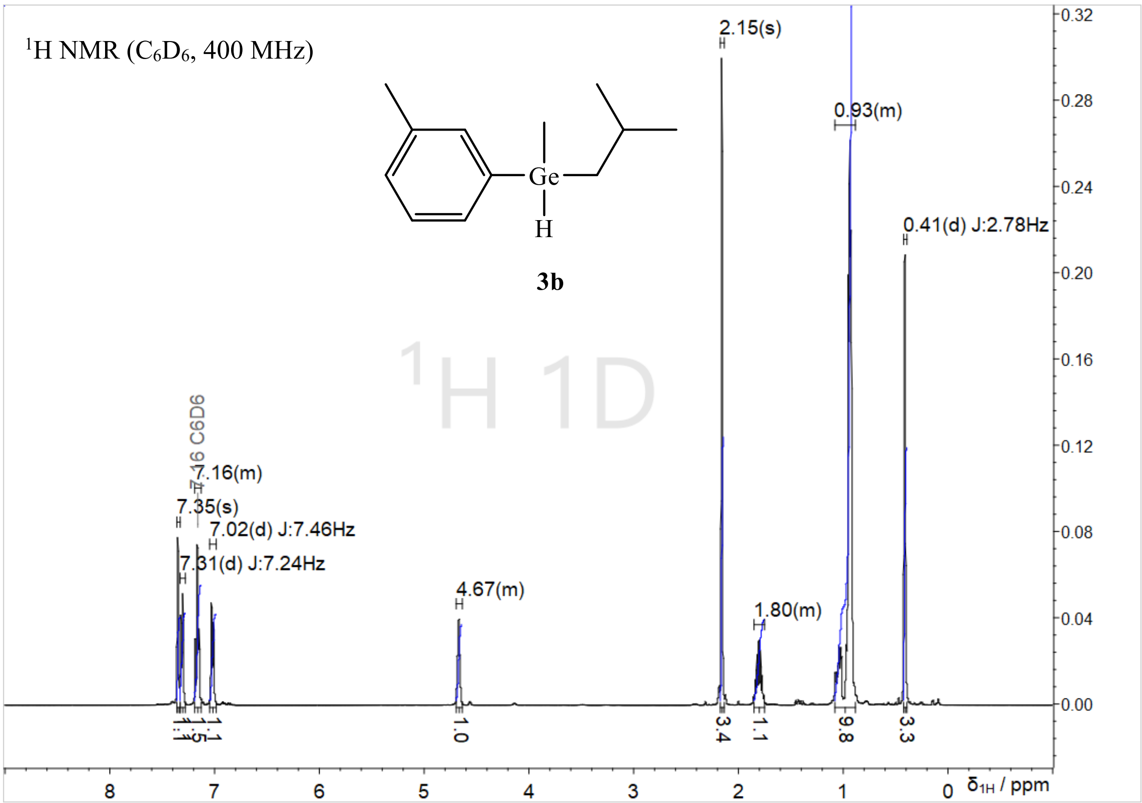

Figure S9. ^{1}H NMR spectrum of **3b** (C_6D_6).

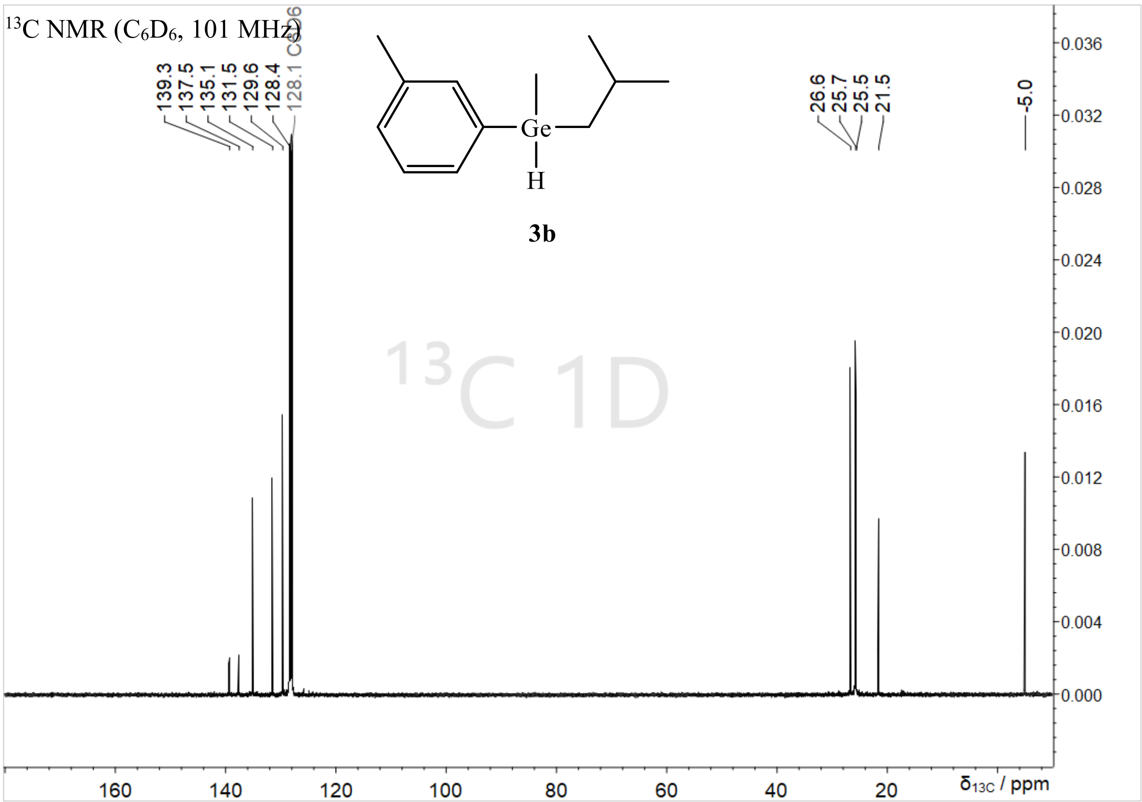

Figure S10. $^{13}C\{^1H\}$ NMR spectrum of **3b** (C_6D_6).

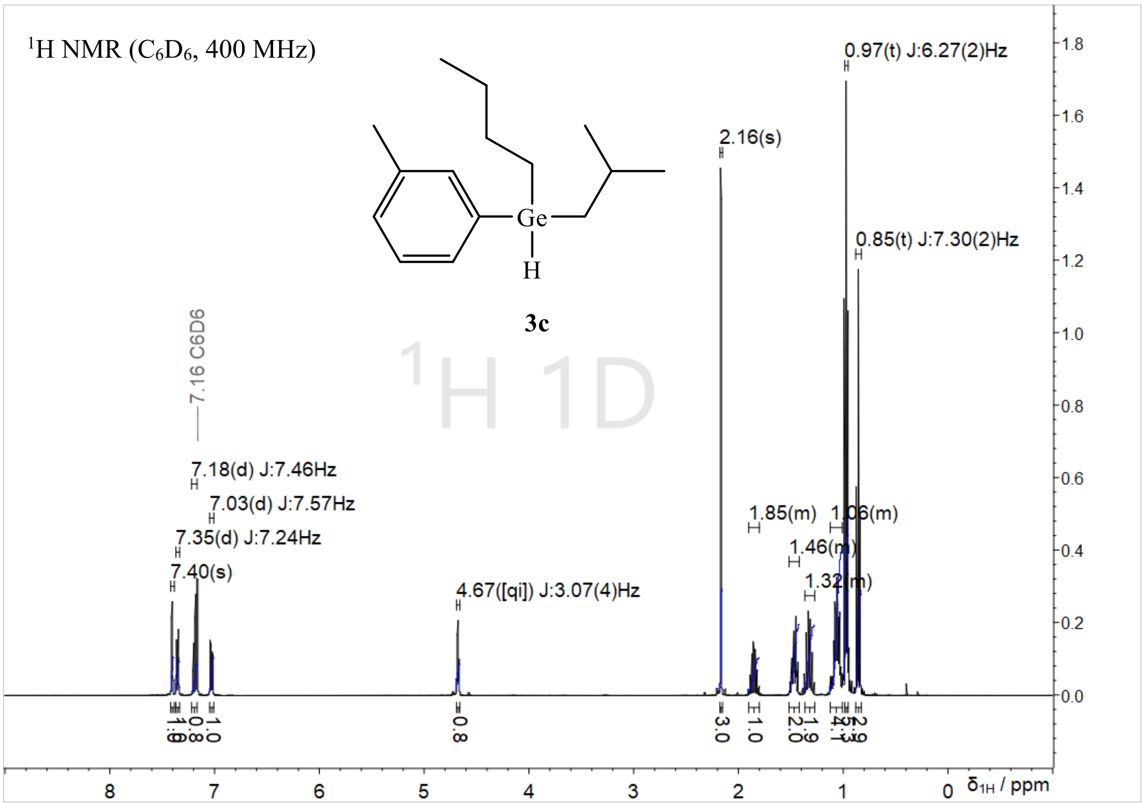

Figure S11. ^{1}H NMR spectrum of **3c** (C_6D_6).

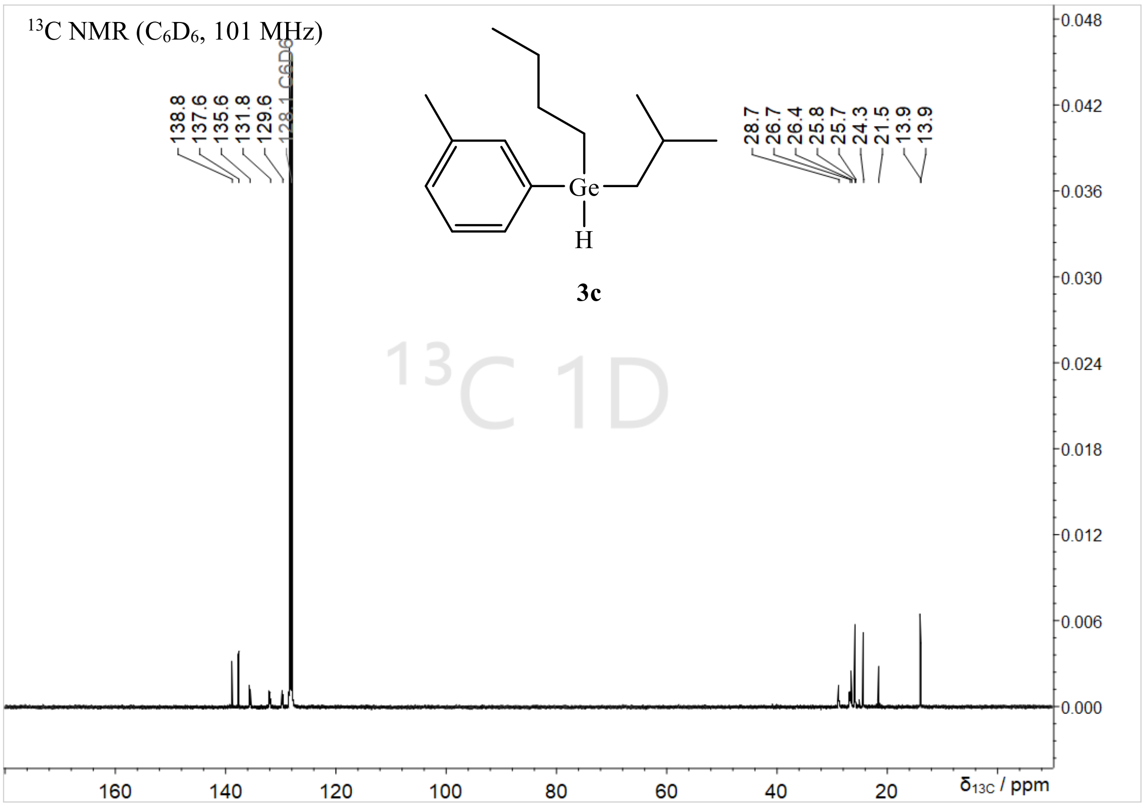

Figure S12. $^{13}C\{^{1}H\}$ NMR spectrum of **3c** (C_6D_6).

2. References:

1. Torvisco, A.; Wolf, M.; Traxler, M.; Gudat, D.; Uhlig, F. New Insights into the Selective and Systematic Preparation of Arylgermanium Hydrides. *Mendeleev Commun.* **2022**, *32*, 22–24.
